# Declarations of conflicts of interest from the editors of the Journal of Global Health – 2021

**DOI:** 10.7189/jogh.11.01008

**Published:** 2021-11-06

**Authors:** Harry Campbell, Ana Marušić, Igor Rudan

We continue the practice of transparency of potential competing interests for us as editors of the Journal of Global Health, following the International Committee of Medical Journal Editors (ICMJE) Recommendations for the Conduct, Reporting, Editing, and Publication of Scholarly work in Medical Journals [1]. As we expect from our authors and reviewers to declare their possible conflicts of interest in relation to the manuscript under consideration for publication in the journal, we also regularly declare our commitments and relations that may influence the editorial review process. When one of the decision-making editors submits an article to the Journal, it is reviewed independently from the editor, who has no access to or influence on the review process and editorial decision-making. This is always indicated in the Competing Interests declaration at the end of the published article.

We declare below the following conflicts of interest, in alphabetical order. We will regularly publish updated declarations when there is a change in editors’ activities and relationships.

## PROFESSOR HARRY CAMPBELL, MD, FRCPE, FFPH, FRSE, FMedSci

### Personal and organizational

Prof. Campbell is employed by the University of Edinburgh, where he holds a position as Professor in the Usher Institute, College of Medicine and Veterinary Medicine. He is also co-Director of the WHO Collaborating Centre in Population Health Research and Training and co-Director of the NIHR Global Respiratory Health Unit. He currently receives research funding from the European Commission (Innovative Medicines Initiative), WHO, UK NIHR and the Baszucki Brain Research Fund. He has received consultancy payments, paid via the University of Edinburgh, from WHO, the Bill and Melinda Gates Foundation, the UK Funding Councils [Research Excellence Framework] and Sanofi in the past 10 years.

### Journal

Prof. Campbell holds a position as the co-Editor in Chief. He occasionally reimburses expenses related to Journal’s work, including travel and consumables. Prof. Campbell is a member of the International Society of Global Health (ISoGH).

### Unpaid positions

Numerous technical advisor appointments to WHO in the past 10 years.

## PROFESSOR ANA MARUŠIĆ, MD, MS, PHD

### Personal and organizational

Prof. Marušić is fully employed by the University of Split School of Medicine, where she holds a tenured position as Professor and Chair of the Department of Research in Biomedicine and Health. She receives funding support for research from the Croatian Science Foundation and the European Commission under Horizon research framewrok. She serves as an external expert for the European Commission.

### Journal

Prof. Marušić holds a position as the Editor in Chief. She occasionally reimburses expenses related to Journal’s work, including travel and consumables. Prof. Marušić is a member of the International Society of Global Health (ISoGH).

### Honorary positions

Honorary Professor, College of Medicine and Veterinary Medicine, University of Edinburgh, Edinburgh, Scotland, UK;

Visiting Professor, School of Medicine, University of Sao Paulo, Sao Paulo, Brazil:

Steering Group, EQUATOR Network;

Co-Chair, Cochrane Scientific Committee;

Research Coordinator, Cochrane Croatia;

Council member, Committee on Publication Ethics (COPE);

Advisory board or editorial board member for a number of medical journals.

## PROFESSOR IGOR RUDAN, MD, DSC, PHD, MPH, FRSE

### Personal and organizational

Prof. Rudan is employed by the University of Edinburgh, where he holds a position as Professor and Co-Director of the Centre for Global Health Research. He is also co-Director of the WHO Collaborating Centre in Population Health Research and Training.

### Journal

Prof. Rudan holds a position as the co-Editor in Chief. He occasionally reimburses expenses related to Journal’s work, including travel and consumables. Prof. Rudan is a member of the International Society of Global Health (ISoGH), and serves as its President.

### Paid positions

Prof. Rudan has numerous technical advisor appointments to WHO, UNICEF and the World Bank.

## References

[1] International Committee of Medical Journal Editors. Recommendations for the conduct, reporting, editing, and publication of scholarly work in medical journals. Disclosure of Financial and Non-Financial Relationships and Activities, and Conflicts of Interest. Available: http://www.icmje.org/recommendations/browse/roles-and-responsibilities/author-responsibilities–conflicts-of-interest.html. Accessed: 5 October 2021.

